# Can standardized patients replace physicians as OSCE examiners?

**DOI:** 10.1186/1472-6920-6-12

**Published:** 2006-02-27

**Authors:** Kevin McLaughlin, Laura Gregor, Allan Jones, Sylvain Coderre

**Affiliations:** 1 University of Calgary, Calgary, Alberta, Canada

## Abstract

**Background:**

To reduce inter-rater variability in evaluations and the demand on physician time, standardized patients (SP) are being used as examiners in OSCEs. There is concern that SP have insufficient training to provide valid evaluation of student competence and/or provide feedback on clinical skills. It is also unknown if SP ratings predict student competence in other areas. The objectives of this study were: to examine student attitudes towards SP examiners; to compare SP and physician evaluations of competence; and to compare predictive validity of these scores, using performance on the multiple choice questions examination (MCQE) as the outcome variable.

**Methods:**

This was a cross-sectional study of third-year medical students undergoing an OSCE during the Internal Medicine clerkship rotation. Fifty-two students rotated through 8 stations (6 physician, 2 SP examiners). Statistical tests used were Pearson's correlation coefficient, two-sample t-test, effect size calculation, and multiple linear regression.

**Results:**

Most students reported that SP stations were less stressful, that SP were as good as physicians in giving feedback, and that SP were sufficiently trained to judge clinical skills. SP scored students higher than physicians (mean 90.4% +/- 8.9 vs. 82.2% +/- 3.7, d = 1.5, p < 0.001) and there was a weak correlation between the SP and physician scores (coefficient 0.4, p = 0.003). Physician scores were predictive of summative MCQE scores (regression coefficient = 0.88 [0.15, 1.61], P = 0.019) but there was no relationship between SP scores and summative MCQE scores (regression coefficient = -0.23, P = 0.133).

**Conclusion:**

These results suggest that SP examiners are acceptable to medical students, SP rate students higher than physicians and, unlike physician scores, SP scores are not related to other measures of competence.

## Background

The objective structured clinical examination (OSCE) is now a commonly used method of assessing clinical competence of medical students and practicing physicians in such areas as history taking, physical examination, diagnostic reasoning and management [1,2]. Compared to written evaluations the OSCE format attempts to increase the examination fidelity by more closely simulating realistic clinical problems or scenarios, and has been shown to have both reliability and construct validity as an evaluation tool [3]. Competence on the OSCE should, therefore, identify students who will subsequently perform well in similar "real life" clinical situations (i.e., competence provides the capacity to perform).

Standardized patients (SP) are increasingly being used in place of 'real' patients for the OSCE as they provide a consistent clinical scenario, thus helping to reduce some of the variability between students' experiences [4]. Many centres are now expanding the role of SP beyond simulated patients by using them to teach clinical skills, an initiative that has been shown to be cost effective [5,6]. As the demand for the OSCE format, and physician time increases, some centres are also employing SP as examiners in addition to their traditional role as patients. In this case the SP are trained to evaluate the students' skills based on a checklist of items for each station. The SP proceeds through the interaction with the student then scores the student based on their observations.

There are both advantages and disadvantages to this approach. A potential methodological advantage to the use of SP may be a reduction in the inter-rater variability in scoring students' performance. This may be the result of a less intimidating environment for students thereby allowing the students to concentrate more on the examination task. A practical advantage to the use of SP would be the reduced need for physician involvement in the examination process. This would alleviate time and scheduling pressures for physicians and may also reduce overall costs of examinations. There is concern, however, that SP may not be adequately trained or experienced to examine students rigorously to achieve the professional standard, or have the background knowledge to identify acceptable variations of students' skills. In addition to this, SP may not have the skills to deliver adequate or appropriate feedback to students during the examination to increase the learning value of the exam for the students. The literature in this area of medical education is limited and conflicting. Some studies have suggested that SP examiners are at least as reliable and 'accurate' as physician examiners in evaluating student performance while others have found SP raters to be inferior to the 'gold standard' physician examiners [7,8]. These conflicting results may partly be explained by the choice of the outcome variable. Less studied, and perhaps of greater importance, is predictive validity of different raters, i.e., how well SP evaluations (and/or physician examiners' evaluations) predict student performance in other areas.

There were three objectives to the present study. The first was to examine students' attitudes towards SP examiners. The second was to determine the correlation between SP examiner and physician examiner scores of medical student performance on OSCE physical exam stations. The third was to compare the predictive validity of scores by SP examiners and physician examiners, using the performance on the summative [problem-solving] multiple choice questions examination (MCQE) as the outcome variable.

## Methods

### Study group

The University of Calgary has a three year undergraduate medical curriculum of which the third year is a clerkship year. During the clerkship year students have a twelve weeks mandatory rotation in internal medicine. At the midpoint of this rotation students have a formative OSCE examination. At the end of the rotation they have a summative problem solving multiple choice (MCQ) examination. This study had a cross-sectional design and involved two consecutive blocks of students rotating through the internal medicine clerkship rotation.

### OSCE and MCQ format

This OSCE comprised nine stations, of which seven stations involved physical examination of a standardized patient. The remaining two stations involved history taking and communication, which were scored by a physician and SP, respectively, and were not included in the study as they evaluated different clinical skills from the physical examination stations. Both blocks of students in this study shared six OSCE stations where a physician examiner was present to evaluate the student and two stations where the SP evaluated the student. One physical examination station was different between the two blocks and was, therefore, not included in the analysis. The physician examiner stations were: precordial examination; respiratory examination; examination of second cranial nerve; assessment of a patient with chest pain; assessment of a patient with dyspnea; and assessment of a patient with liver disease. The SP examiner stations were: examination of the knee; and examination of the spleen. Both the physician and SP examiners had a checklist of historical and/or physical exam components that students were expected to elicit or demonstrate. The individual components were totaled to provide an overall score for this station, expressed as a percentage. The mean scores for the six physician examiner stations and the two SP examiner stations were calculated for each student.

The summative MCQ examination is a problem solving examination with both high reliability and content validity, the latter provided by a published examination blueprint. For each student in the study the score on the MCQ examination was recorded.

### Questionnaire to evaluate students' attitudes toward SP examiners

Following completion of the OSCE the students were asked to rate their degree of agreement with five statements about the use of SP examiners. Student responses were based on a 5 point Likert scale ranging from strongly disagree to strongly agree. The statements were:

***1. I did not know what I was expected to do on this station***.

*2. I found this station more stressful than stations with a physician examiner*

*3. Standardized patients give feedback as effectively as physicians*

*4. Standardized patients are not sufficiently trained to judge examination skills of a clerk*

***5. I would like to see more stations where the standardized patient is the examiner***.

### Data analysis

Student attitudes towards SP examiners were expressed as proportions after the Likert scale rating was dichotomized. The decision to dichotomize these scores was made a priori as these scores are ordinal. Strongly disagree and disagree were combined and considered as "disagreement". Strongly agree and agree were combined and considered as "agreement". A neutral response (answer 3 on the Likert scale) was considered a missing data point. The correlation between the mean scores for physician examiner stations and SP examiner stations was evaluated using Pearson's correlation coefficient. The mean scores for physician examiner and SP examiner stations were compared using a two-sample test of variance and a two-sample t-test. Effect size was calculated using the method described by Cohen [9]. Multiple linear regression was used to study the relationship between the mean scores for physician examiner stations, SP examiner stations and the continuous dependent variable summative MCQ result. The regression model tested for interaction between the physician examiner and SP examiner scores. All statistical tests were two-sided and a p value of <0.05 was considered statistically significant. All analyses were performed using STATA 7.0 software (Stata Corporation, College Station, Texas).

## Results

### Students' attitudes towards SP examiners

Fifty-two students participated in this study. No student reported not knowing what to expect for the SP examiner station. A minority of students (4.8%) considered the SP examiner station more stressful than the physician examiner station. More than half of the students (52.9%) felt that SP examiners were as good as physician examiners in giving feedback. Less than one third of students (31.6%) felt that SP examiners were not sufficiently trained to judge the examination skills of a clerk and approximately one third of students (36.4%) of students would like to have seen more SP examiner stations.

### Rating of students' competency by SP and physician examiners

The mean score (± SD) for student performance as judged by SP examiners was 90.4% (± 8.9) compared to 82.2% (± 3.7) for physician examiners. This difference was statistically significant (P < 0.0001) with a large effect size (d = 1.5). The correlation coefficient for SP examiner and physician examiner scores for each student was 0.4 (P = 0.003). Considering the dependent variable of summative MCQ result, there was no significant interaction between SP scores and physician scores. Physician examiner scores were significantly and positively related to the summative MCQ result. For every 1% increase in the physician score for the OSCE the summative MCQ score [± 95% CI] increased by 0.88% [0.15, 1.61] (P = 0.019). There was no significant relationship between SP scores for the OSCE and the summative MCQ score (regression coefficient = -0.23, P = 0.133).

## Discussion

We report the results of a cross-sectional study comparing SP examiners to physician examiners for a third year medical student internal medicine OSCE. Our results show that SP are acceptable as examiners to students in this type of examination. We showed a weak but significant correlation between SP examiners' and physician examiners' scores, although SP examiners tended to score students higher than physician examiners. Using performance on the formative multiple choice MCQ exam as an endpoint, physicians' scores on the OSCE had predictive value, whereas SP examiner scores did not.

Why do SP examiners score students higher than physician examiners? One possible explanation is that SP examiners may simply want to give students a higher mark, or at least the benefit of the doubt, as this favours a more pleasurable student-SP encounter (physicians are, of course, not immune to this as they may have prior knowledge of the students and can also expect future encounters, both of which may introduce a 'halo effect' into evaluation). While this may partially explain the 'determination bias' in the SP examiners score that inflates the students' scores when compared to physician examiner scores, it is unlikely to be the sole reason as a systematic inflation should mean scores that are higher than physician examiners' but retain predictive validity. A more likely explanation is that as a result of their limited training and background knowledge, SP may not be able to distinguish between students with surface knowledge and those with deep understanding of the topic. They may, therefore, inconsistently overestimate (± underestimate) students' competence at performing the required task. SP do not have the experience of seeing many students at different levels perform the same task over many years, as do physician examiners, and therefore do not have the same standard for comparison. By contrast, it has previously been shown that SP examiners do not overestimate ability in more 'generic' skills, such as communication [10].

SP documentation of examinee performance is already an integral part of several high-stakes examinations, including the USMLE. Opinions differ, however, as to who should evaluate the various components of the examinee performance. In a recent review on this topic as it relates to a high-stakes examination (Educational Commission for Foreign Medical Graduates' Clinical Skills Assessment), Whelan et al propose a hybrid form of evaluation in which each attribute is evaluated by the person best suited to evaluate. They suggest that aspects of communication are best evaluated by the patient (or the replacement for the patient) whereas problem solving skills are best evaluated by content experts, i.e., physicians [11]. This study offers some support to the argument that clinical skills, such as physical examination skills, are better evaluated by content experts than SP.

### Study limitations

This study has several important limitations. Firstly, in this study the SP and physicians examined different stations. This introduces the possibility of performance bias related to the specific stations. To address this we plan to compare SP and physician examiner evaluations of students on the same stations in future studies. By this head-to-head comparison we may be able to identify stations or tasks where SP could replace physician examiners and those where SP examiner scores are less valid. Another limitation is that this study evaluated the predictive validity of a formative test of competency compared to a summative test of competency. Ideally a test of 'performance' should be used as the outcome measure and should be congruent with the OSCE in evaluating behaviour-based performance rather than higher cognitive function that is evaluated in the MCQ.

### Implications

It is unlikely that SP will completely replace physician examiners in the medical student evaluation process. However, with the growing number of medical students and physicians' increasingly busy schedules, educators may have to develop new ways to continue the evaluation process with limited physician involvement. One solution is to limit the OSCE to a formative evaluation or teaching tool, although many would argue against subordinating this reliable and valid evaluation with high fidelity to evaluations with lower fidelity, such as written evaluations [12]. While students appear to find SP acceptable as examiners, the challenge will be to improve the predictive validity of SP evaluations. In order to do this, SP may require additional training to discern between students with surface and deep knowledge [13]. If this is unsuccessful or unfeasible they may have a more limited role as examiners on specific types of stations or they may function in combination with physicians to evaluate different components of a single task.

### Future research

Further studies are needed to evaluate the impact of additional training of SP on the ability to discern between students with surface and deep knowledge. Further studies are also needed to clearly define the potential role of SP examiners as a replacement for or addition to physician examiners.

## Conclusion

Our results suggest that while SP are acceptable as examiners to students, their rating of student competence is higher than that of physician examiners and is not predictive of rating on other tests of competence.

## Competing interests

The author(s) declare that they have no competing interests.

## Authors' contributions

All authors contributed to the design, data collection and paper preparation.

## Pre-publication history

The pre-publication history for this paper can be accessed here:


